# Overall survival and morbidity are not associated with advanced age for cytoreductive surgery and hyperthermic intraperitoneal chemotherapy: a single centre experience

**DOI:** 10.1515/pp-2022-0202

**Published:** 2023-04-11

**Authors:** Ernest Cheng, Raphael Shamavonian, Jasmine Mui, Raymond Hayler, Josh Karpes, Ruwanthi Wijayawardana, Shoma Barat, Nima Ahmadi, David L. Morris

**Affiliations:** Department of Surgery, Peritonectomy and Liver Cancer Unit, St George Hospital, Kogarah, NSW, Australia; St George Hospital Clinical School, University of New South Wales, Kogarah, NSW, Australia

**Keywords:** age factors, cytoreductive surgery, hyperthermic intraperitoneal chemotherapy, pseudomyxoma peritonei, survival analysis

## Abstract

**Objectives:**

Cytoreductive surgery with hyperthermic intraperitoneal chemotherapy (CRS/HIPEC) has enabled better prognosis for patients with peritoneal surface malignancies. However, in older age groups, short -and long-term outcomes are still perceived as poor. We evaluated patients aged 70 and over and determine if age is a predictor of morbidity, mortality and overall survival (OS).

**Methods:**

A retrospective cohort analysis was performed on CRS/HIPEC patients and categorised by age. The primary outcome was overall survival. Secondary outcomes included morbidity, mortality, hospital and incentive care unit (ICU) stay and early postoperative intraperitoneal chemotherapy (EPIC).

**Results:**

A total of 1,129 patients were identified with 134 aged 70+ and 935 under 70. There was no difference in OS (p=0.175) or major morbidity (p=0.051). Advanced age was associated with higher mortality (4.48 vs. 1.11 %, p=0.010), longer ICU stay (p<0.001) and longer hospitalisation (p<0.001). The older group was less likely to achieve complete cytoreduction (61.2 vs. 73 %, p=0.004) and receive EPIC (23.9 vs. 32.7 %, p=0.040).

**Conclusions:**

In patients undergoing CRS/HIPEC, age of 70 and above does not impact OS or major morbidity but is associated with increased mortality. Age alone should not be a limiting factor in selecting CRS/HIPEC patients. Careful multi-disciplinary approach is needed when considering those of advanced age.

## Introduction

Peritoneal surface malignancies are a manifestation of late-stage intra-abdominal malignancy, where the cancer has disseminated to the abdominal peritoneal surface [1]. Historically, patients with such widespread disease were considered palliative, with few curative surgical options available. However, the development of cytoreductive surgery (CRS) with hyperthermic intraperitoneal chemotherapy (HIPEC), pioneered by Dr Sugarbaker in the 1990s, has revolutionised treatment for peritoneal surface malignancies [2, 3]. Advancements in this field have led to significantly improved long-term outcomes, particularly for patients with appendiceal cancers with peritoneal spread [4, 5]. The median survival of patients undergoing CRS/HIPEC remains variable, ranging from 30 to 248 months depending on the primary histopathology [4], [5], [6], [7]. The addition of additional methods of delivering chemotherapy such as early postoperative intraperitoneal chemotherapy (EPIC) as further improved patient survival outcomes [8], [9], [10].

One caveat in performing maximally invasive surgery such as CRS/HIPEC is the risk of increased morbidity and limited survival benefit with increasing age. Older age is often associated with frailty, increased co-morbidities and longer recovery time which is a barrier to invasive and extensive abdominal surgery [11], [12], [13]. Hence, maximally invasive procedures tend to be reserved for the younger population aged less than 70 years old. CRS/HIPEC is considered a morbid procedure, with long operating times, extensive resections, and high risk of serious post-operative complications [14]. There is thought to be increased morbidity and mortality in operating on older patients and this may negate any long-term survival benefits gained from CRS/HIPEC [15, 16]. However, this perception is not adequately demonstrated in the literature with mixed results regarding morbidity, mortality and survival outcomes [17, 18]. All patients who are made eligible for CRS/HIPEC meet a stringent selection criterion which should be regardless of age.

The need to evaluate operative risk in older patients is paramount, given that life expectancy is rising worldwide and is expected reach 78 years of age by 2050. It is predicted that the proportion of people aged 60 and over will triple to 21 % in this same period [19]. This rapid shift towards an aging population correlates to a proportional increase in older patients who will require major abdominal surgery. The current literature on CRS/HIPEC in patients with advanced age is limited. There are conflicting results mainly from studies with small sample sizes and a paucity of long-term data reporting survival outcomes [17, 18]. Particularly for those aged 70 and over, the safety and survival outcomes are still unclear [15, 17, 20, 21]. As an experienced high-volume CRS and HIPEC centre, our present study aims help establish a consensus and provide data to guide decision making on patients 70 and older undergoing this operation.

## Materials and methods

### Study design and patient selection

A retrospective analysis of patients that underwent both CRS and HIPEC between January 1996 and March 2022 was performed using a prospectively maintained database at the Peritonectomy Surgery Unit at St George Hospital, Kogarah, New South Wales, Australia. Patients were categorised dichotomously according to their age at the time of their index surgery. Group 1 consisted of patients aged under 70 and Group 2 with patients aged 70 or over.

The classification of patients as “elderly”, “old age” or “advanced age” in the literature remains arbitrary. Previous studies frequently define this cut-off at age 65, however, patients aged 70, 75 and even 80 undergoing major abdominal surgery have been an increasing focus in the literature [22], [23], [24], [25]. The United Kingdom Office of National Statistics claims that the definition of “older age” should be based on a remaining life expectancy of 15 years [26]. In Australia, where our unit is based, the average male is expected to live to 81.2 years and female to 85.3 years according to the Australian Institution of Health and Welfare [27]. We therefore determined that a cut-off age of 70 is most appropriate for this study. We also recognise that this cut-off should vary and be adjusted accordingly to different cultural, social and healthcare settings around the world.

Signed informed consent was obtained from all patients for the collection of clinical data. This study was approved by the local Ethics Committee as part of an ongoing prospective observational investigation.

### Pre-operative work up

All patients underwent a comprehensive standardised pre-preoperative work up inclusive of history, physical examination, blood tests and imaging. This included a full panel of bloods including tumour markers and computed tomography (CT), ultrasound, magnetic resonance imaging (MRI) and positron electron tomography (PET) imaging where relevant. All patients were discussed in a multi-disciplinary meeting which included surgeons, medical oncologists, radiologists, and allied health members. Eligibility for CRS and HIPEC was assessed on an individual basis based upon pathology, co-morbidities and functionality. Age alone was not a reason for exclusion from surgery.

### Cytoreductive surgery

CRS was performed based on the principles described by Sugarbaker et al. [3]. A laparotomy was performed, and a peritoneal cancer index (PCI) was obtained to grade the volume of disease. This was calculated by dividing the abdomen into 13 regions and assigning a score between 0 – 3 depending on macroscopic tumour size yielding a final score out of 39 [1]. Resection of the primary tumour and all involved visceral abdominal organs and parietal surfaces was then performed. Following this, the completeness of cytoreduction (CC) score was assessed macroscopically ranging from 0 to 3. CC-0 indicated no residual disease, CC-1 indicated remaining disease less than 2.5 mm, CC-2 indicated remaining disease ranging between 2.5 to 25 mm and finally CC-3 indicated remaining disease over 25 mm.

### Hyperthermic intraperitoneal chemotherapy

HIPEC was performed after CRS using the open “Coliseum” technique with the appropriate chemotherapy agent heated to 41.5 °C. The length of HIPEC time was dependent on the type of chemotherapeutic agents used.

### Early postoperative intraperitoneal chemotherapy (EPIC)

EPIC aims to target microscopic disease and CRS/HIPEC by maximising disease exposure to chemotherapeutic agents prior to the development of post-operative adhesions. This was administered to patients with appendiceal or colorectal tumours via a peritoneal catheter placed intraoperatively. 5-Fluororacil chemotherapy at 650mg/m2 with 50 mEq of sodium bicarbonate was administered for 23 h, and then drained. This was repeated up to five times depending on several clinical criteria. This included the absence of leakage around drain sites, no major organ failure or sepsis, normal intraabdominal pressures, adequate urine output and ability to tolerate additional intra-abdominal fluid.

### Morbidity and mortality

All postoperative complications were recorded in concordance with the Clavien–Dindo Classification (CDC) [28]. CDC grades of I or II defined minor morbidities and grades III to V were considered major morbidities. A mortality was classified as any death that occurred either during the index admission or within 90 days of the index operation. The total days spent in ICU was calculated based on length of ICU stay from index admission. Total length of hospital stay was calculated from date of index admission to date of discharge from hospital.

Routine follow up was conducted every three months for at least 5 years including physical examination, tumour markers and CT imaging.

### Statistical analysis

All statistical analyses were performed using IBM SPSS software version 24 (IBM corporation, New York, USA). Normally distributed quantitative data were analysed using Student’s T tests and the Mann-Whitney U test when appropriate. Categorical variables were compared using Pearson’s Chi square test. Survival outcomes were estimated using the Kaplan–Meier method with OS calculated from date of index operation to last follow-up visit or date of death. Cox proportional hazard regression was utilised to assess the effect of age, PCI, CC score, tumour type and complications on OS. Both uni-variant and multi-variant analysis were performed on all patients and on patients aged 70 and above. A 95 % confidence interval with a p-value of <0.05 indicated statistical significance.

## Results

A total of 1,129 patients were identified with a mean age of 54.8 years (standard deviation (SD) 13.3) and a median of 55.8 years (range 14.4–84.3). 995 patients were under 70 years of age with a mean of 52.2 years (SD 11.7) and a median of 53.6 years (range 14.4–69.9). 134 patients were aged 70 or older with a mean of 74.68 years (SD 3.97) and a median of 73.5 (range 70.0–84.3). The most common primary diagnoses were appendiceal cancers consisting of 46.9 % of all cases, followed by colorectal (32.4 %), mesothelioma (7.62 %) and ovarian (6.91 %). A total of 6.11 % had other pathologies inclusive of gastric, urothelial, breast and adenocarcinoma of unclear origins. Out of the 31.6 % of patients who received EPIC, patients aged 70 and above were less likely to receive EPIC (23.9 vs. 32.7 % p=0.040). Patient characteristics are summarised in Table 1.

**Table 1:** Comparative clinicopathological and operative demographics data for patients undergoing CRS and HIPEC for peritoneal surface malignancies according to age.

	Group 1: Age under 70	Group 2: Age 70 or over	p-Value
Demographics data			
Total number of patients			
Gender	995	134	
Male, n (%)	419 (42.1)	66 (49.3)	0.117
Female, n (%)	576 (57.9)	68 (50.7)	
Age, years (mean SD)	52.2 (11.73)	74.68 (3.97)	<0.001
Primary tumour			
Appendix, n (%)	467 (46.9)	63 (47.0)	0.296
Colorectal, n (%)	317 (31.9)	49 (36.6)	
Mesothelioma, n (%)	76 (7.64)	10 (7.46)	
Ovarian, n (%)	69 (6.93)	9 (2.65)	
Others, n (%)	66 (6.63)	3 (2.24)	
HIPEC chemotherapy			
Mitomycin-C	537	79	
Oxaliplatin	301	40	
Cisplatin	73	9	
Cisplatin and mitomycin-C	78	6	
Others	6	0	
Operative data			
PCI score			
1–20, n (%)	602 (60.5)	73 (54.5)	0.209
21–39, n (%)	392 (39.5)	60 (45.5)	
CC-score			
CC 0, n (%)	726 (73.0)	82 (61.2)	0.004
CC 1+, n (%)	269 (27.0)	52 (38.8)	
Post-operative outcomes			
Major morbidity			
CD score 3–5, n (%)	338 (34.0)	57 (42.5)	0.051
Mortality, n (%)	11 (1.11)	6 (4.48)	0.010
ICU length of stay, mean (SD)	4.26 (7.64)	7.72 (15.6)	<0.001
Total days length of stay, mean (SD)	26.13 (20.2)	37.2 (33.1)	<0.001
EPIC			
Yes, n (%)	325 (32.7)	32 (23.9)	0.040
No, n (%)	670 (67.3)	102 (76.1)	

n, number; SD, standard deviation; CRS, cytoreductive surgery; HIPEC, hyperthermic intraperitoneal chemotherapy; PCI, peritoneal cancer index; CC, completeness of cytoreduction; CD, Clavien–Dindo score.

A total of 59.8 % of patients had a PCI between 1-20 and 40.2 % had PCI between 21-39 with a mean PCI score of 17.9 (SD 12.1). Both groups had similar volume of disease in terms of PCI (p=0.209). Complete cytoreduction (CC=0) was achieved in 71.6 % of all patients. Patients under the age of 70 were more likely to have complete cytoreduction compared to those aged 70 and over (73.0 vs. 61.2 % p=0.004). The perioperative factors are summarised in Table 1.

### Short term outcomes; morbidity, mortality and length of stay

A total of 28.0 % patients experienced no postoperative complications. Minor complications (CD I or II) were observed in in 37.0 % of all patients. The overall rate of major morbidities (CD III to V) was 35.0 % and there was no significant difference between the two groups (p=0.051). Excluding mortalities (CD V), major morbidities (CD III and IV) occurred in 32.9 % of Group 1 and 38.1 % of Group 2 patients (p=0.137) (Table 1).

Mortality rate (CD grade V) consisted of 1.51 % of all patients. There were 11 mortalities in Group 1 and 6 in Group 2. Patients aged 70 and over were significantly more likely to experience a mortality (p=0.010). However, no clear preoperative predictive factor of mortality was identified in all 17 patients. Two patients aged over 80 died with intra-abdominal sepsis one month after their initial surgery. One patient died from hospital acquired pneumonia and two from aspiration pneumonia. One patient experienced necrotising fasciitis secondary to an enterotomy and failed to recover from sepsis. There were no deaths in patients in Group 2 intraoperatively or immediately post-operatively. Causation of inpatient death in Group 1 patients was similar to those in Group 2, with a combination of postoperative sepsis, aspiration or hospital acquired pneumonia.

Both ICU and hospital length of stay was longer in the older patient group. Mean ICU length of stay was 7.72 days in Group 2 compared to 4.26 days in Group 1 (p<0.001). Mean total length of hospital stay was 37.2 days in Group 2 compared to 26.1 days in Group 1 (p<0.001).

### Long term outcomes; overall survival

**Figure 1:**
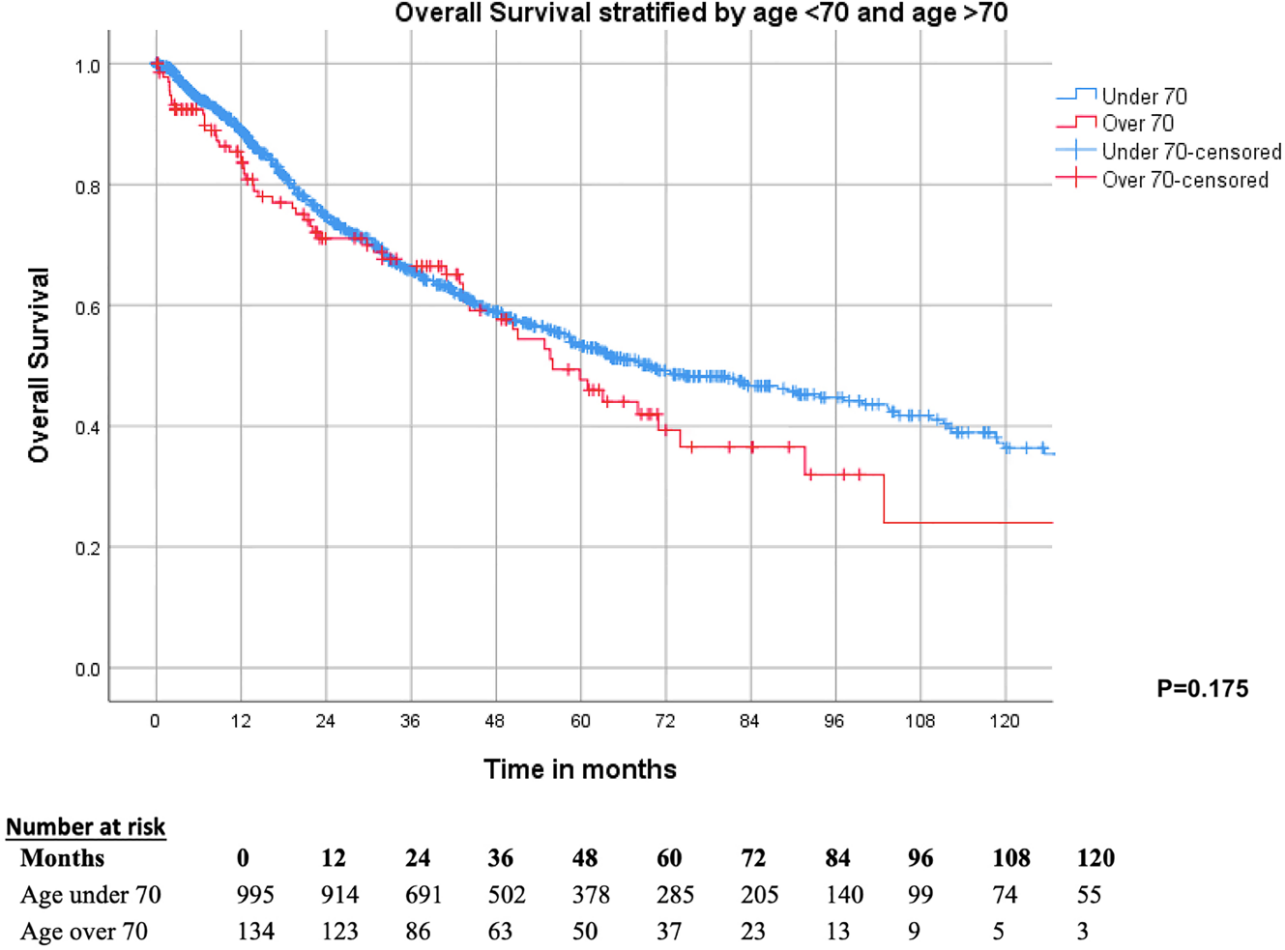
Kaplan–Meier survival curve demonstrating no difference between OS in patients aged 70 and above and those aged under 70.

Kaplan–Meier analyses stratified by age demonstrated similar survival outcomes between Group 1 and Group 2, with no difference in OS (p=0.175) (Figure 1). The 1-, 3-, 5- year OS was 89.1 , 66.2 and 53.6 % for Group 1 and 83.7 , 66.5 and 48.4 % for Group 2, respectively (Table 2). The median OS was 67.9 months (CI 95 % 55.9–79.9) for all patients, 69.8 months (95 % CI 55.5–84.0) for Group 1 and 56.0 (95 % CI 44.3–67.8) for Group 2.

**Table 2:** Survival rates comparing both groups.

Months	Group 1: Age under 70 (n=995), %	Group 2: Age over 70 (n=134), %
12	89.1	83.7
24	75.0	71.0
36	66.2	66.5
48	59.2	59.7
60	53.6	48.4

Operative and post-operative variables were analysed with uni- and multi-variable Cox regression analyses for all patients and for Group 2 patients alone. For all patients, variables included age, PCI, CC score, significant morbidity, and EPIC. When adjusted for co-variants, age was not a significant factor in OS (HR 0.948 95 % CI 0.75–1.34, p=0.961). Patients who experienced major complications demonstrated worse OS with a HR of 0.437 (95 % CI 0.35–0.547, p<0.001). Patients who received EPIC resulted in improved OS (p<0.001) (Table 3). For those aged 70 and over, PCI, CC score and morbidity were not associated with lower OS. The use of EPIC in this patient group continued to show improved OS with a HR of 0.503 (95 % CI 0.257–0.984, p=0.045) (Table 4).

**Table 3:** Univariate and multivariate analyses of all patients.

	Univariable, HR (95 % CI)	p-Value	Multivariable, HR (95 % CI)	p-Value
Age				
<70	Reference			
≥70	1.22 (0.917–1.61)	0.175	0.948 (0.757–1.34)	0.961
PCI				
0–20	Reference			
21–39	1.24 (1.03–1.50)	0.027	1.08 (0.849–1.37)	0.534
CC score				
CC=0	Reference			
CC 1 +	1.47 (1.21–1.79)	<0.001	1.31 (1.02–1.68)	0.034
Complication CD 3–4				
No	Reference			
Yes	1.51 (1.25–1.83)	<0.001	1.54 (1.58–1.30)	<0.001
Received EPIC				
No	Reference			
Yes	0.470 (0.377–0.585)	<0.001	0.437 (0.350–0.547)	<0.001

PCI, peritoneal cancer index; CC, completeness of cytoreduction; CD, Clavien–Dindo; EPIC, early post-operative intraperitoneal chemotherapy.

**Table 4:** Predictors of survival in patients aged 70 and over.

	Univariable, HR (95 % CI)	p-Value	Multivariable, HR (95 % CI)	p-Value
PCI				
0–20	Reference			
21–39	1.36 (8.05–2.32)	0.247	1.412 (0.688–2.896)	0.347
CC score				
CC=0	Reference			
CC 1 +	1.22 (0.717–2.081)	0.463	0.898 (0.430–1.874)	0.774
Complication CD 3–4				
No	Reference			
Yes	1.188 (0.699–2.019)	0.525	1.134 (0.629–2.045)	0.676
Received EPIC				
No	Reference			
Yes	0.513 (0.263–1.004)	0.051	0.503 (0.257–0.984)	0.045

PCI, peritoneal cancer index; CC, completeness of cytoreduction; CD, Clavien–Dindo; EPIC, early post-operative intraperitoneal chemotherapy.

## Discussion

Our results demonstrated no statistically significant difference in OS despite increased mortality in the older population. While this has similarly been reported in the literature (Table 5), there are also several other studies which have demonstrated worse OS outcomes for older patients [29], [30], [31], [32], [33], [34], [35], [36], [37], [38]. However, many of these studies consisted of small sample sizes and were undertaken in small peritonectomy units, thus, results may be confounded by inexperience or low case volumes for operating on older patients. Our unit undertakes an average of 3–5 CRS/HIPEC procedures a week, for the last 20 years without discriminating patients based on age. As a result, our surgeons are adept at performing CRS/HIPEC and have mastered the learning curve, thus minimising operation times and rates of complications over time. This subsequently has had an impact on our outcomes with operating on older patients with lower physiological reserve. Therefore, we show promising results with no difference in long term OS for patients aged 70 and older.

**Table 5:** Summary of studies comparing survival outcomes for CRS/HIPEC patients stratified by age.

Author and year	No. of older patients	Cut off age, years	Survival outcomes of older patients	Comparison to younger group
Macri et al. 2011 [32]	11	65		No difference
Tabrizian et al. 2013 [34]	20	65	Median survival 21.2 months	No difference
Spiliotis et al. 2014 [36]	9	70	1-,3-year survival 75 and 27.7 %	Worse
Beckert et al. 2015 [29]	29	70	Median survival 21 months	No difference
Huang et al. 2015^a^ [31]	124	65	Median survival 43 months	No difference
Wong et al. 2017 [15]	18	65	1-,3-,5- year OS 79.4 , 59.6, 59.6 %	No difference
Katai et al. 2017 [16]	14	70	5- year OS 41.3 %	Worse
Arslan et al. 2018 [47]	13	65	2-year OS 77 %	No difference
Ezzedine et al. 2020 [21]	30	65	1-,3-,5- year OS 85 %, 74 % and no reach	No difference
Zambrano-Vera et al. 2021 [15]	19	65	Median survival 33.5 months	Worse
Zhou et al. 2021 [38]	25	65	1-, 3-, 5-year OS of 51.4 , 27.2 and 16.3 %	Worse
Laks et al. 2022 [48]	35	70	64.6 months median survival	No difference

^a^Same database used in this study.

The impact of the learning curve on OS is further demonstrated by a study by Votanopoulos et al. who compared survival outcomes at his centre in the first decade of performing CRS/HIPEC with current practice outcomes for patients aged over 70 years [39]. Their study demonstrated a significant increase in the median survival and a reduction in mortality for patients who underwent CRS/HIPEC in more recent years. Thus, they concluded that high volume operating, and the learning curve does make a difference for survival outcomes in patients over the age of 70. We advocate that in high-volume peritonectomy centres, age should not be a limiting factor for offering CRS/HIPEC.

Another reason for the discord between our survival data and the data represented in the literature may relate to the diversity of primary malignancies and extent of peritoneal disease in these studies. For example, in Spiliotis et al.’s study, 30 patients over the age of 70 had a 27.7 % 3-year survival rate [36]. However, 80 % of their patients had colorectal and ovarian primary malignancy, which has less favourable outcomes compared to appendiceal tumours which make up the bulk primary malignancy of our study. Henceforth, as a high-volume centre with a large focus on appendiceal cancers, we recognise our findings may not be applicable to all CRS/HIPEC centres.

Our data also demonstrated no significant difference in morbidity when comparing patients over the age of 70 with those 70 or younger who underwent CRS/HIPEC. The reported postoperative major morbidity for patients of advanced age undergoing CRS/HIPEC varies significantly ranging between 18–71 % [17, 40, 41]. We reported that 38.1 % of patients over 70 experienced a major morbidity (Clavien–Dindo grade III or IV) which was not significantly different to patients under 70 (32.9 %). On the other hand, Turgeon et al., found increased complication rates for patients over 65 but only for those with invasive histology. A meta-analysis by Tao et al., reported higher complication rates for those aged 70 and above, but for studies with a cut-off of 65, no difference was observed [18]. Interestingly, Tao et al. reported no difference in hospital length of stay between age groups. Major complications and hospital length of stay often go hand in hand; however, we observed an increase in ICU and overall hospital length of stay for older patients. This phenomenon can be due to slower healing responses exhibited in older patients and thus a longer recovery from the initial surgery itself as well as subsequent complications [42]. Managing these patients in a multi-disciplinary team setting with geriatrician input perioperatively is therefore paramount.

Interestingly, the older cohort was less likely to receive complete cytoreductions and treatments with EPIC. Both are associated with prolonging OS but can result in increased postoperative complications [43], [44], [45]. This raises the question of the merits in pursuing complete cytoreduction or administration of EPIC in older patients, especially if OS remains unchanged. However, there are no previous studies comparing older patients and the outcomes of receiving EPIC. Therefore, the decision for EPIC in older patients should be highly individualised, based on several patient, perioperative and tumour factors.

In terms of mortality, our older group had a comparatively higher rate of 4.48 % compared to 1.11 %. Our detailed examination into the mortality of each patient aged over 70 did not identify any specific common age-related factor. This rate is higher than the overall reported mortality of 1.1–2.4 % in the literature for patients of all ages undergoing CRS/HIPEC [14, 46]. However, when observing the mortality rate for patients over 65 in isolation, this ranges from 1.2-5.4 % which mirrors our results [18]. This increased mortality rate is important to consider and convey to older patients being considered for CRS/HIPEC but should be individualised to the patient given the wide disparity in risk.

### Limitations

This study’s outcomes are limited by the individualised nature of the selection process for patients undergoing CRS/HIPEC. Rather than utilising specific prognostication tools for the older patients, our unit takes a multi-disciplinary approach in evaluating both patient and tumour related factors to determine who is best suited for CRS/HIPEC. Therefore, we are unable to specify predictive or confounding factors that have impacted the significance of our results when comparing outcomes between the two groups. Furthermore, we did not propensity match our two group of patients; however, the similarity in preoperative characteristics and the relative size of our two groups significantly reduces this confounding bias. Additionally, we lack data on how many patients we turned down and the reasons why. Quality-of-life impacts and endpoints in older patient group could have added further value to this study. The retrospective nature of this study intrinsically is limiting with selection bias.

## Conclusions

Controversy and doubt will continue to exist when subjecting patients of advanced age to maximally invasive surgery, but like all treatments, an optimal balance of risk and benefit must be achieved. In this study, we demonstrate that age alone does not limit survival and morbidity, but impacts mortality, when pursuing CRS/HIPEC. Our results demonstrate an overall feasibility in operating on older patients, however, we stress that these results may not be translatable to less experienced or lower volume centres. Therefore, patients over 70 should be referred to dedicated experienced high-volume CRS/HIPEC centres for optimal management. In carefully selected older patients, outcomes can be comparable with younger patients undergoing CRS/HIPEC.

## Highlights

– Morbidity and overall survival are not affected in patients 70 aged over undergoing CRS/HIPEC
– Mortality, length of hospital and intensive care unit stay is increased with advanced age
– Patients of this age group require careful selection and should be managed at experienced centres
